# Comparison of five equations in describing the variation of leaf area distributions of *Alangium chinense* (Lour.) Harms

**DOI:** 10.3389/fpls.2024.1426424

**Published:** 2024-07-04

**Authors:** Linli Deng, Ke He, Karl J. Niklas, Zhuyue Shi, Youying Mu, Peijian Shi

**Affiliations:** ^1^ Department of Applied Mathematics, College of Science, Nanjing Forestry University, Nanjing, China; ^2^ Architectural Design and Research Institute, Shenzhen University, Shenzhen, China; ^3^ School of Civil Engineering and Architecture, Xiamen University of Technology, Xiamen, China; ^4^ School of Integrative Plant Science, Cornell University, Ithaca, NY, United States

**Keywords:** Akaike information criterion, leaf area distribution, Lorenz curve, model comparison, nonlinear regression

## Abstract

Previous studies have validated a performance equation (PE) and its generalized version (GPE) in describing the rotated and right-shifted Lorenz curves of organ size (e.g., leaf area and fruit volume) distributions of herbaceous plants. Nevertheless, there are still two questions that have not been adequately addressed by prior work: (i) whether the PE and GPE apply to woody plant species and (ii) how do the PE and GPE perform in comparison with other Lorenz equations when fitting data. To address these deficiencies, we measured the lamina length and width of each leaf on 60 *Alangium chinense* saplings to compare the performance of the PE and GPE with three other Lorenz equations in quantifying the inequality of leaf area distributions across individual trees. Leaf area is shown to be the product of a proportionality coefficient (*k*) and leaf length and width. To determine the numerical value of *k*, we scanned 540 leaves to obtain the leaf area empirically. Using the estimated *k*, the leaf areas of 60 A*. chinense* saplings were calculated. Using these data, the two performance equations and three other Lorenz equations were then compared and assessed using the root-mean-square error (RMSE) and Akaike information criterion (AIC). The PE and GPE were found to be valid in describing the rotated and right-shifted Lorenz curves of the *A. chinense* leaf area distributions, and GPE has the lowest RMSE and AIC values. This work validates the GPE as the best model in gauging variations in leaf area of the woody species.

## 1 Introduction

Prior work has shown that even small differences in the morphological traits of leaves can affect the photosynthetic and respiration rates (Zirbel et al., 2017) and that leaf (lamina) area is a critical functional trait for a large number of plant species (Westoby, 1998), providing the maximum information on plant growth and resource utilization (Hodgson et al., 1999). Therefore, accurately measuring the leaf area and the extent to which it varies across and within species is important to understand plant ecology and evolution.

The Montgomery equation (ME) assumes a proportional relationship between leaf area and the product of leaf length and leaf width (Montgomery, 1911). Previous work has shown that the ME can effectively predict the area of even intricately shaped lamina (Shi et al., 2019a, b; Yu et al., 2020; Schrader et al., 2021). In addition, Yu et al. (2020) found that the ME is superior to other mathematical models using leaf length and width based on data drawn from 15 species of vines which included some complex leaf shapes. However, prior studies delving into the utility of the ME have used computer recognition methods to identify the leaf maximum width perpendicular to the leaf length axis. This approach is difficult under field research conditions to accurately and non-destructively identify leaf maximum width. Fortunately, a recent study has shown a negligible difference between the use of computer recognition methods and manual measurements defining leaf width by simply connecting two leaf boundary landmarks (Mu et al., 2024).

To assess variations in leaf area, prior work has turned to using the mathematics of the Lorenz curve, which is commonly used in economics to describe the inequality of income distributions (Lorenz, 1905; Kakwani, 1977) by plotting the cumulative proportion of household income against the cumulative proportion of the number of households. When the income distributions across households are absolutely equal, the Lorenz curve overlaps with the straight line passing through (0, 0) and (1, 1), i.e., *y = x*. In turn, the Gini coefficient (GC) is used to quantify the extent to which the Lorenz curve deviates from the line of absolute equality, which equals twice the area formed by the Lorenz curve and the line of absolute equality. The closer the GC is to 0, the more equal the income distribution tends to be. More recently, the GC has been used in some botanical studies due to its ability to quantify the inequality of urban green space, plant, and plant organ size distributions (Taylor and Aarssen, 1989; Matlack, 1994; Metsaranta and Lieffers, 2008; Chen et al., 2014; Nero, 2017; Huang et al., 2023; Lian et al., 2023)—for example, the unevenness of leaf size distributions can be quantified by the GC because the Lorenz curve can quantify the relationship between the cumulative proportion of leaf area and the cumulative proportion of the number of leaves.

The accuracy of this approach has been investigated using a performance equation (PE) and its generalized version (GPE) to fit the rotated and right-shifted Lorenz curves of the organ size distributions of herbaceous species (Huang et al., 2023; Lian et al., 2023; Shi et al., 2024; Wang et al., 2024)—for example, Wang et al. (2024) compared the PE and the GPE with two other performance equations based on a temperature-dependent square root equation proposed by Ratkowsky et al. (1983) and found that the GPE had the best goodness of fit and lowest Akaike information criterion (AIC). In recent years, researchers have compared Lorenz equations in quantifying the inequality or non-uniformity of the size distributions of abiotic as well as biotic quantities (Sarabia, 1997; Sarabia et al., 1999; Sitthiyot and Holasut, 2023). However, prior work has not compared the two performance equations that describe the rotated and right-shifted Lorenz equations with other Lorenz equations, perhaps because the parameters of the Lorenz equations are estimated by minimizing the residual sum of squares (RSS) between the observed and predicted cumulative proportions of plant (or organ) size, whereas the parameters of the PE and the GPE are estimated by minimizing the residual sum of squares (RSS) between the observed and predicted cumulative proportions of plant (organ) size when rotated and right-shifted. Consequently, there is a need to develop a method that can directly compare the two performance equations and other Lorenz equations to evaluate which equation is more accurate when quantifying the inequality of plant (or organ) size distributions.

In the present study, we measured the leaf length (*L*) and width (*W*) data of 752 leaves from 60 *Alangium chinense* (Lour.) Harms saplings. The leaf area (*A*) was determined using the ME, which assumes a proportional relationship between *A* and *LW*. In addition, 540 leaves were scanned to determine the *A*, *L*, and *W* of the 540 leaves empirically. We employed five models to fit the rotated and right-shifted Lorenz curves of individual leaf area distributions (i.e., the cumulative proportion of leaf area per sapling vs. the cumulative proportion of the number of leaves per sapling). Our goals were to address two questions: (i) Can the PE and GPE be used to describe the rotated and right-shifted Lorenz curve of leaf area distributions of woody species? (ii) Are the two performance equations superior to other Lorenz equations used in nonlinear regression? If true, this approach provides a non-destructive method to quantify leaf area and inequalities in organ size distributions of both woody and herbaceous species, which could provide deeper insights into extant (and possibly extinct) plant ecology and evolution.

## 2 Materials and methods

### 2.1 Leaf sampling information and data acquisition

**Figure 1:**
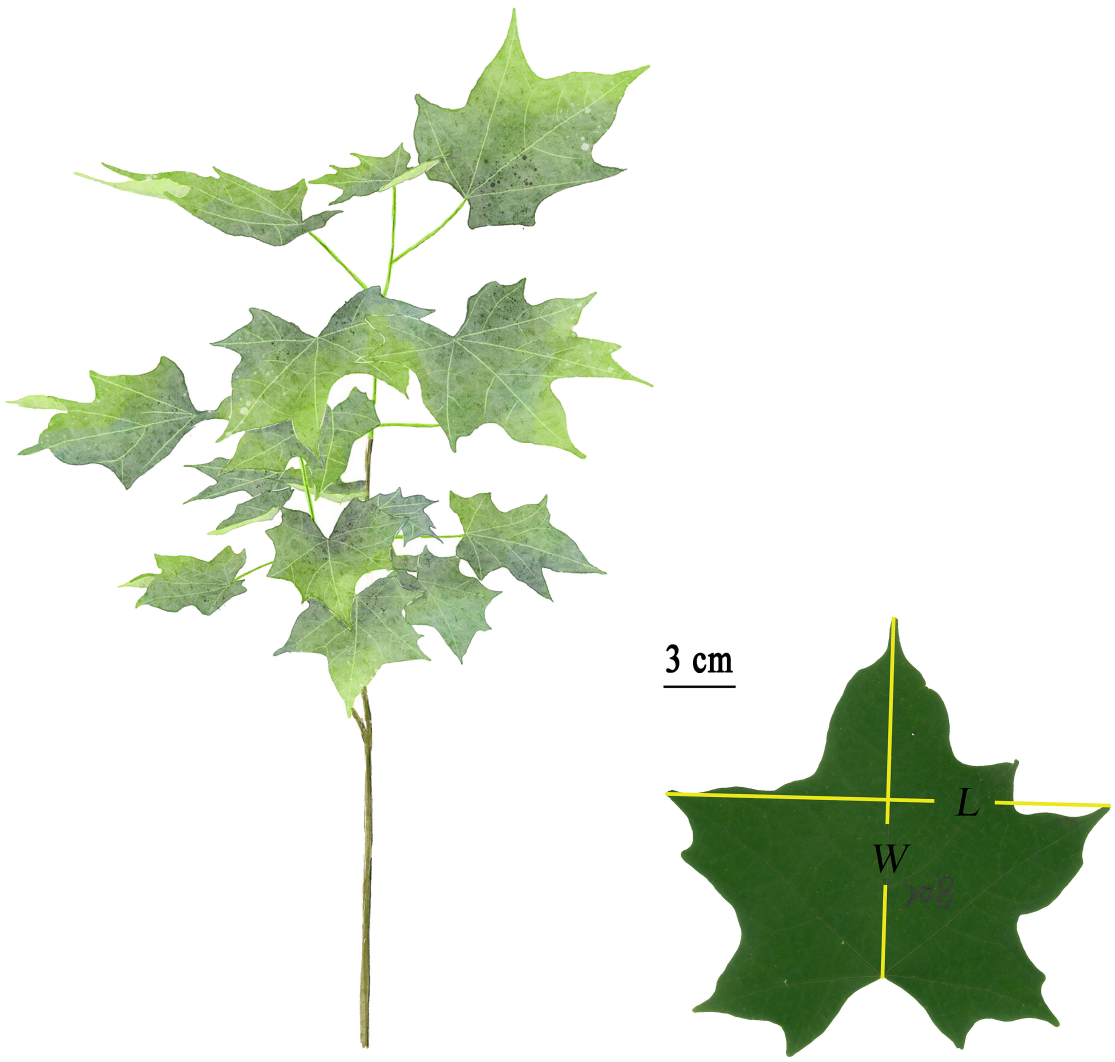
Freehand drawing of the above-ground part of *A. chinensis* and definition of leaf length (*L*) and width (*W*). *L* was defined as the distance between leaf tip and the connection point between the lamina and the petiole, and *W* was defined as the distance between the two leaf apices.

Dataset 1: A total of 540 fresh, mature, and undamaged leaves of *A. chinense* were sampled from the middle canopy of 10 saplings with a diameter at breast height (DBH) of 2 to 7 cm growing in the Nanjing Forestry University campus, Nanjing, China (32°4′48″ N, 118°49′12″ E) on July 31, 2019. Leaves in groups of four were placed in plastic self-sealing bags (28 cm × 20 cm) to avoid tissue dehydration and taken to the laboratory within 1 h. Laminae were scanned to bitmap images at 600-dpi resolution using a photo scanner (Aficio MP 7502; Ricoh, Tokyo, Japan). ImageJ software (https://imagej.nih.gov/ij/index.html) was used to measure the area (*A*), length (*L*), and width (*W*) of each leaf (see online **Supplementary Table S1**). *W* was defined by the maximum distance between two lobe apices (**Figure 1**).

Dataset 2: On October 17, 2023 and on May 18, 2024, we randomly selected 60 A*. chinense* saplings, each with at least nine leaves, to provide a sample size of not less than 1.5 times the minimum data required to perform nonlinear regression analyses to estimate the regression parameters of Equation 4 (see below for details). The above-ground height of the saplings ranged between 30.6 and 136.4 cm, with a mean ± standard error of 81.3 ± 28.5 cm. We measured the *L* and *W* of each leaf of the 60 saplings without removing the leaves from the branches. A total of 752 leaves from the 60 saplings were examined (see online **Supplementary Table S2**).

### 2.2 Estimate of leaf area based on leaf length and width

Montgomery (1911) proposed a formula to estimate the leaf area (*A*) of corn (*Zea mays* Linn.) using the product of a proportionality coefficient (*k*) and leaf length (*L*) and width (*W*):


(1) A=kLW

which is denoted as Montgomery equation (ME), and *k* is referred to as Montgomery parameter (MP) hereinafter. To normalize *A*, Equation 1 was log-transformed:


(2) log(A)=c+log(LW)

where *c* is a constant to be estimated. It is apparent that *c* = log*k*. Equation 2 was used to fit the 540 empirical observations of *A* vs. *LW* and thereby determine the numerical value of *c*.

### 2.3 Equations to describe the rotated and right-shifted Lorenz curve

The cumulative proportions of leaf area per sapling vs. the cumulative proportion of the number of leaves per sapling were rotated counterclockwise by 135° around the origin and shifted to the right by a distance of $\sqrt{2}$ . Previous studies have used two performance equations for fitting the size frequency distributions of leaf size and fruit volume data of herbaceous plants (Huang et al., 2023; Lian et al., 2023; Wang et al., 2024):

(i) The performance equation, denoted as PE, which was initially used to describe the jumping distance of green frogs at different body temperatures (Huey, 1975; Huey and Stevenson, 1979):


(3) $$y=c\left(1-e^{-K_{1}\left(x-x_{1}\right)}\right)\left(1-e^{K_{2}\left(x-x_{2}\right)}\right)$$

In the context of our study, *x* and *y* represent the rotated and right-shifted cumulative proportion of the number of leaves per sapling and the cumulative proportion of leaf area per sapling, respectively; *c*, *K*_1_, and *K*_2_ are constants to be estimated; *x*_1_ and *x*_2_ are equal to 0 and $\sqrt{2}$ , respectively (Lian et al., 2023; Wang et al., 2024).

(ii) The generalized performance equation, denoted as GPE, which increases the flexibility of data fitting by adding two parameters, *a* and *b*, to PE (Lian et al., 2023):


(4) $$y=c{\left(1-e^{-K_{1}\left(x-x_{1}\right)}\right)}^{a}{\left(1-e^{K_{2}\left(x-x_{2}\right)}\right)}^{b}$$

In the context of our study, *x* and *y* represent the rotated and right-shifted cumulative proportion of the number of leaves per sapling and the cumulative proportion of leaf area per sapling, respectively; *x*_1_, and *x*_2_ are also equal to 0 and $\sqrt{2}$ (Lian et al., 2023).

Equations 3 and 4 were used to evaluate the performance of Lorenz curve fitting. In terms of the latter, Sarabia (1997) proposed a highly flexible parameter family of Lorenz curves based on the generalized Tukey λ distribution for fitting Lorenz curves:


(5) $$y_{l}=\left(1-\text{λ}+\text{η}\right)x_{l}+\text{λ}x_{l}^{a_{1}+1}-\text{η}\left[1-{\left(1-x_{l}\right)}^{a_{2}+1}\right]$$

In the context of our study, *x_l_* and *y_l_* represent the cumulative proportion of the number of leaves per sapling and the cumulative proportion of leaf area per sapling, respectively, and λ, η, *a*_1_, and *a*_2_ are constants to be estimated, where $a_{1}\geq 0$ , $a_{2}+1\geq 0$ , $\text{η}a_{2}+\text{λ}\leq 1$ , λ≥0 , and $\text{η}a_{2}\geq 0$ . Equation 5 is denoted as SarabiaE hereinafter.

Sarabia et al. (1999) proposed a general method for building parametric-functional families of Lorenz curves generated from an initial Lorenz curve, which satisfies some regularity conditions:


(6) $$y_{l}=x_{l}^{\text{γ}}{\left[1-{\left(1-x_{l}\right)}^{\text{α}}\right]}^{\text{β}}$$

In the context of our study, *x_l_* and *y_l_* represent the cumulative proportion of the number of leaves per sapling and the cumulative proportion of leaf area per sapling, respectively, and α, β, and γ are constants to be estimated, where 0<α≤1 , β≥1 , and γ≥0 . Equation 6 is denoted as SCSE hereinafter.

Importantly, the existing parameter function forms of Lorenz curve are not suitable for extreme inequalities in size distributions. To fit sample data with typical convex segments better in a Lorenz curve, a universal function for fitting Lorenz curves was proposed (Sitthiyot and Holasut, 2023):


(7) $$\begin{array}{c} y_{l}=\left(1-\text{ρ}\right)\left[\left(\frac{2}{P+1}\right)\left(\frac{x_{l}-\text{δ}}{1-\text{δ}}\right)\right]\\ +\text{ρ}\left[\left(1-\text{ω}\right){\left(\frac{x_{l}-\text{δ}}{1-\text{δ}}\right)}^{P}+\text{ω}\left\{1-{\left\{1-\left(\frac{x_{l}-\text{δ}}{1-\text{δ}}\right)\right\}}^{\frac{1}{P}}\right\}\right] \end{array}$$

when $x_{l}>\text{δ}$ ; $y_{l}=0$ , when $x_{l}\leq \text{δ}$ . Here *x_l_* and *y_l_* represent the cumulative proportion of the number of leaves per sapling and the cumulative proportion of leaf area per sapling, respectively, and δ, ρ, ω, and *P* are constants to be estimated, where 0≤δ<1 , 0≤ρ≤1 , 0≤ω≤1 , and P≥1 . Equation 7 is denoted as SHE hereinafter.

We rotated and right-shifted the three foregoing Lorenz equations [i.e., Equations 5-7] to evaluate the performance of the PE and GPE with these three Lorenz equations in describing the rotated and right-shifted data of the cumulative proportion of leaf area per sapling vs. the cumulative proportion of the number of leaves per sapling.

### 2.4 Data fitting and model evaluation

We used the PE, GPE, SarabiaE, SCSE, and SHE equations to fit the empirical data after the data were rotated counterclockwise by 135° and shifted to the right by a distance of $\sqrt{2}$ . The Nelder–Mead optimization algorithm (Nelder and Mead, 1965) was then used to minimize the fitting criteria for nonlinear regression and the residual sum of squares (RSS) between empirical and predicted *y*-values to estimate the parameters of each model. To better evaluate the goodness of fit of nonlinear regression, the root-mean-square error (RMSE) was used to measure the prediction accuracy. The smaller the RMSE, the higher the prediction accuracy of the model. The “fitLorenz” function in the “biogeom” package (version 1.4.3; Shi et al., 2022) based on statistical software R (version 4.3.3; R Core Team, 2024) was used to fit the leaf area distribution:


(8) $$\text{RMSE}=\sqrt{\text{RSS}/n}$$

where *n* represents the number of leaves per sapling. The Akaike information criterion (AIC) that compared to the adjusted coefficient of determination is frequently recommended in nonlinear regression to balance the goodness of fit and the model constructive complexity (Spiess and Neumeyer, 2010). The model with the smallest AIC is considered as the optimal model. Paired *t*-test at a significance level of 0.05 was used to test the significance of differences in the RMSE or AIC values between any two of the five equations.

## 3 Results

**Figure 2:**
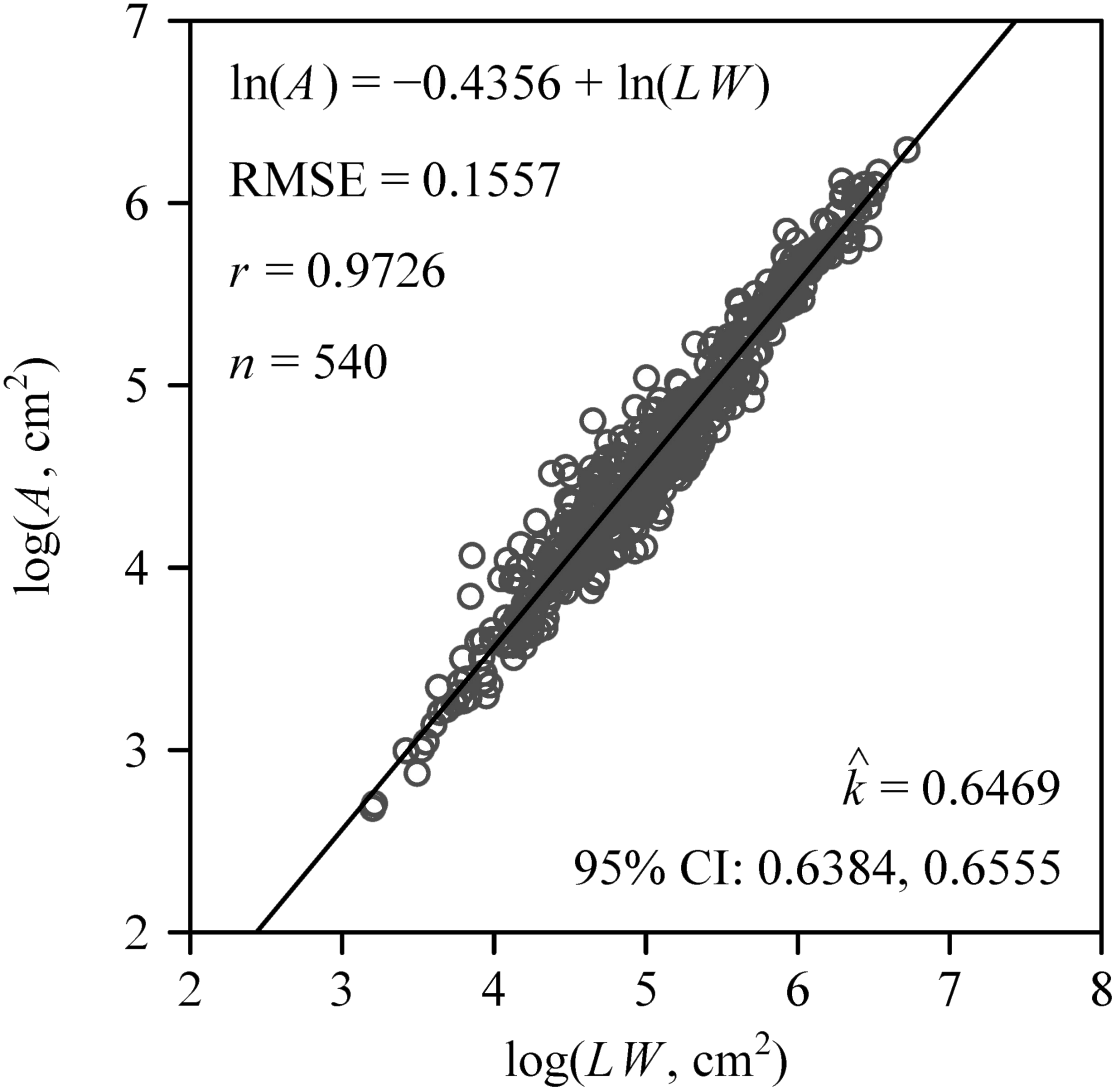
Results of fitting the Montgomery equation that assumes a proportional relationship between *A* and *LW*, which represent the leaf area, length, and width, respectively. Here RMSE represents the root-mean-square error; *r* represents the correlation coefficient; *n* represents the number of samples; $\hat{k}$ represents the estimated value of the Montgomery parameter, i.e., the proportionality coefficient of the Montgomery equation; 95% CI represents 95% confidence intervals of the Montgomery parameter based on 3,000 bootstrap replicates.

The number of leaves per sapling ranged between nine and 25, with a mean ± standard error of 12.5 ± 3.8. The Montgomery equation (ME) was validated in estimating *A* by multiplying the leaf length and width by the estimated *k*. The correlation coefficient (*r*) between *A* and *LW* was 0.9726, and the RMSE (see Equation 8) of linear regression was 0.1557 (**Figure 2**). The estimated value of the proportionality coefficient *k* was 0.6469. Using this value of *k*, the area of each leaf of the 60 saplings was obtained. The total leaf area per sapling ranged between 564 and 2,875 cm^2^, with a mean ± standard of 1,391 ± 548 cm^2^. The mean leaf area of each sapling ranged between 56 and 183 cm^2^, with a mean ± standard error of 111 ± 28 cm^2^.

**Figure 3:**
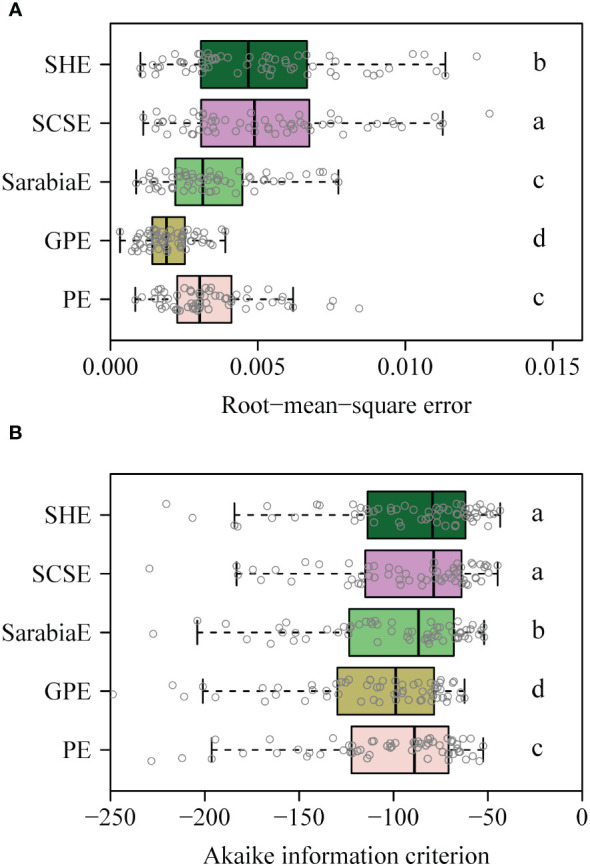
**(A, B)** Boxplot of the root-mean-square error between any two of the five equations (i.e., PE, GPE, SarabiaE, SCSE, and SHE) for 60 datasets. Paired *t*-test was used to determine the significance of differences between any two equations at the 0.05 significance level, and different letters represent a significant difference of any two equations. The vertical solid line in each box represents the median.

**Figure 4:**
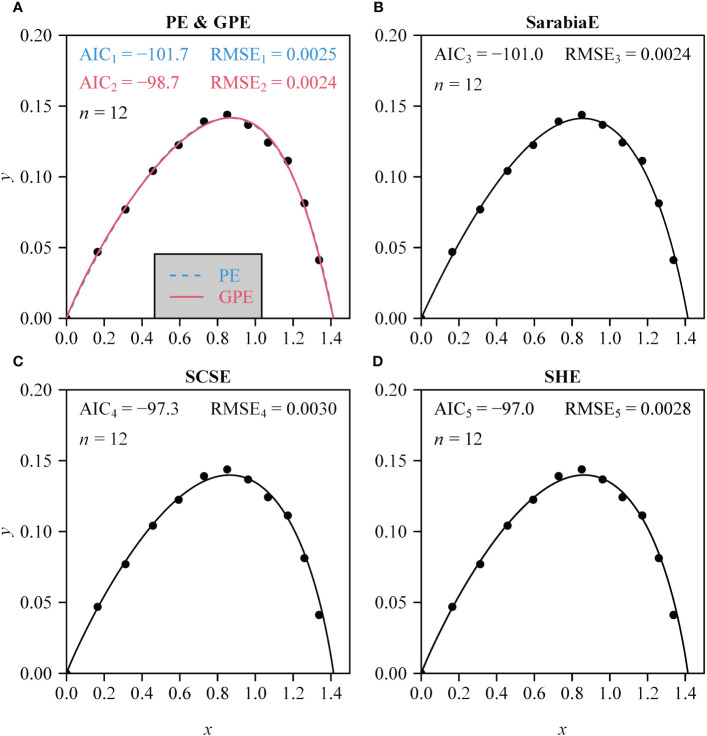
**(A–D)** Comparison of the observed and predicted data of the rotated and right-shifted Lorenz curve for the leaf area distribution of a representative individual sapling of *A. chinensis*. Data points represent observations; curves represent predicted values. Specifically, in **(A)**, the blue curve represents the estimated PE, and the red curve represents the estimated GPE. *n* represents the number of leaves on this individual sapling; AIC represents the Akaike information criterion of the corresponding nonlinear equation in each panel; RMSE represents the root-mean-square error.

The PE, GPE, SarabiaE, SCSE, and SHE fitted the rotated and right-shifted data of the leaf area distributions of the 60 saplings well, with RMSE values <0.013. However, the GPE had the lowest RMSE and AIC values among the five equations. The SarabiaE and PE worked the second best and were better than SCSE and SHE (**Figure 3**). The RMSE and AIC values of GPE were both significantly lower than those of the other equations (*p* < 0.05). **Figure 4** shows the fitted results of the five equations to the rotated and right-shifted data of leaf area distribution of one sapling example.

## 4 Discussion

The goals of this paper were to compare equations describing the inequality of plant size distributions and to evaluate which among the contending equations perform the best. It is important, therefore, to review the criteria used to evaluate the equations investigated in this study. A secondary but equally important goal was to demonstrate the utility of the approach taken in this and other studies using the Montgomery equation. These goals are discussed in the following two sections.

### 4.1 Criteria to evaluate competitive equations

When evaluating the superiority of competitive nonlinear models, researchers often use the root-mean-square error (RMSE) and Akaike information criterion (AIC) (Akaike, 1998). RMSE can be used not only to measure the prediction accuracy of a model on continuous data but also to measure the average degree of deviation between the predicted and observed values. A smaller RMSE indicates a more accurate prediction of the model. The AIC is based on the concept of entropy and can balance the complexity of the estimated model with the goodness of fit of the estimated model. It is apparent that increasing the number of free parameters in a model can improve the goodness of fit and enhance the flexibility of a model to fit the data. Thus, the value of the likelihood function increases as the model complexity increases, which leads to a smaller AIC. Conversely, when the increased rate of the likelihood function slows down, it can lead to an increase in AIC, i.e., AIC encourages the goodness of fit but avoids an overfitting, if the model is complex. We argue that the priority of model selection should focus on the lowest AIC value.

In the present study, the performance equation (PE) and its generalized version (GPE) are validated based on 60 empirical datasets (i.e., the leaves removed from 60 saplings). The RMSE and AIC values of GPE were significantly smaller than those of the four other contending equations. Specifically, GPE worked best, and PE worked second best in that it had the second lowest AIC value. Both were superior to the SarabiaE, SCSE, and SHE. Clearly, the GPE has more parameters than PE. However, PE contains fewer parameters that can reduce the probability of overfitting. Although GPE has the lowest RMSE and AIC values, PE is still recommended due to its fewer parameters and validity in fitting the rotated and right-shifted data. Only for extremely right-skewed distributions can SHE perform better than the two performance equations (Shi et al., 2024).

### 4.2 The general applicability of the Montgomery–Lorenz Performance approach

A pivotal goal of this paper was to evaluate a very specific and targeted approach to quantifying the size frequency distributions of plants and plant organs (in this case, the leaves of saplings). The approach presented here, which can be called the Montgomery-Lorenz-Performance (MLP) approach, involves a threefold mathematical methodology. The Montgomery equation allows us to quantify leaf area (as well as many other variables of interest) non-destructively once the Montgomery parameter (i.e., the proportionality coefficient of ME) is determined (Schrader et al., 2021), which requires a comparatively small number of samples from a population of leaves. The Lorenz curve, in tandem with the Gini coefficient, as a methodology, allows us to quantify the evenness/unevenness of any size frequency distribution. Lastly, the performance equation and its generalized form allow us to evaluate the reliability of the quantification of size frequency distribution evenness/unevenness.

At issue is whether this overall approach has general applicability. We argue that the evidence indicates that the methodology has general applicability because the mathematics underlying each component of the overall approach has been validated here and elsewhere both in terms of its mathematical rigor and its empirical predictive ability. Because of its analytical rigor and empirical robustness, the methodology presented here and elsewhere has application to many aspects of ecological enquiry, not just to plant organs, because the metrics used to measure “size” (e.g., length, width, volume, and biomass) can be applied to any cellular form of life, extant or extinct.

## 5 Conclusions

We compared the two performance equations (i.e., PE and GPE) with three other Lorenz equations (i.e., SarabiaE, SCSE, and SHE) in describing the rotated and right-shifted data of the cumulative proportion of leaf area per sapling vs. the number of leaves per sapling for a representative broad-leaved plant (i.e., *Alangium chinensis*). The five equations were found to be valid in describing the rotated and right-shifted Lorenz curve of the leaf area distributions of this tree species. However, the GPE was found to be superior, and PE worked the second best compared with the other three equations based on the comparison of their AIC values. This refined method provides a general protocol for quantifying the inequality of any organic size distribution, which is of great significance in analyzing the allocation of organismic resources.

## Data availability statement

The original contributions presented in the study are included in the article/**Supplementary Material**. Further inquiries can be directed to the corresponding author.

## Author contributions

LD: Formal analysis, Methodology, Writing – original draft. KH: Investigation, Writing – original draft. KN: Formal analysis, Methodology, Writing – review & editing. ZS: Investigation, Writing – review & editing. YM: Investigation, Writing – review & editing. PS: Formal analysis, Methodology, Writing – review & editing.
